# Examining Virtual Reality Interventions for Social Skills in Children with Autism Spectrum Disorder: A Systematic Review

**DOI:** 10.1007/s10803-025-06741-y

**Published:** 2025-02-05

**Authors:** Yücel Altın, Özge Boşnak, Ceyda Turhan

**Affiliations:** 1 Republic of Türkiye Ministry of National Education, Istanbul, Türkiye; 2 Uludağ University, Bursa, Türkiye

**Keywords:** Autism spectrum disorder, Virtual reality, Social skills, Intervention

## Abstract

Autism Spectrum Disorder (ASD) is a neurodevelopmental disorder that is characterized by limitations in social communication and interaction, self-repetitive behaviors, and the presence of limited interests. The prevalence of ASD, which typically emerges in the first years of life, is increasing at an alarming rate due to multiple factors, including the broadening of diagnostic criteria, heightened public awareness, and more frequent diagnoses among women and adults. Over the years, experts have invested considerable time and effort in developing educational scenarios for children with ASD. However, they have faced challenges replicating certain scenarios—such as emergencies, crowded public transportation, or restaurant environments—because recreating these exact conditions in real-world settings is difficult or cost-prohibitive. This has consequently compelled experts to seek out supplementary intervention methods that are more suitable and accessible. Virtual reality (VR), which has the capacity to integrate the physical and virtual realms, represents one such alternative intervention method. In this study, a systematic review of studies employing VR technology in social skills interventions for individuals with ASD was conducted, and 31 studies were included. The findings indicate the potential benefits of VR applications focusing on the social skills of individuals with ASD. Additionally, this research elucidates the limitations of the studies and offers suggestions for future research.

## Introduction

Autism Spectrum Disorder (ASD) is a neurodevelopmental disorder that is characterized by limitations in social communication and interaction, self-repetitive behaviors, and the presence of limited interests (American Psychiatric Association [APA], 2022). The prevalence of ASD, which typically emerges in the first years of life, is increasing at an alarming rate due to multiple factors, including the broadening of diagnostic criteria, heightened public awareness, and more frequent diagnoses among women and adults (Russell et al., 2022). A report published by the U.S. Centers for Disease Control and Prevention (CDC) indicates that one in 36 children is affected by ASD (Maenner et al., 2023). This represents an increase from the previous CDC report, published in 2021, which concluded that one in 44 children is affected by ASD (Maenner et al., 2021).

One of the distinguishing characteristics of ASD is persistent deficits in social communication and social interaction across multiple contexts (APA, 2022). Difficulties in initiating and maintaining social communication, interaction, and reciprocal engagement are among the core features of children with ASD. Therefore, targeting the domain of social skill development is critically important for the success of children with autism (Silveira-Zaldivar et al., 2021).

In their 1988 study, Morgan and Jenson defined social skills as comprising both verbal and nonverbal behaviors that facilitate mutually beneficial social interactions. Goodwin (1999) defined social skills as abilities that enable individuals to function independently in society and enhance their quality of life. As can be gleaned from the definitions provided, social skills serve to enhance and enrich the quality of individuals' lives. This is achieved by enabling them to interact effectively with others in their daily lives, develop friendships, participate in leisure activities, and become integrated into a group (Turhan & Vuran, 2015). Consequently, these experiences are of paramount importance for individuals with ASD (Vuran, 2007).

Experts have historically utilized various methods to teach social skills to children with ASD, including video modeling, behavioral skills training, social stories, peer- and sibling-mediated interventions, antecedent interventions, manualized curricula, pivotal response training, milieu teaching, naturalistic interventions, reinforcement, script fading, discrete trial training, and self-management (Radley et al., 2020). However, they have faced challenges due to the significant time and effort required to create diverse instructional scenarios and the difficulty posed by certain non-replicable scenarios (Dyson et al., 2019). Consequently, experts have been compelled to identify supplementary intervention strategies that are more suitable and accessible. Virtual reality (VR), which has the capacity to integrate the tangible and the virtual, represents one such alternative intervention method. An increasing number of studies have demonstrated the efficacy of VR in treating children with ASD (Amaral et al., 2018; Babu et al., 2017; Bozgeyikli et al., 2016; Hocking et al., 2022; Kim et al., 2024; Maskey et al., 2019; Moon & Ke, 2023; Schmidt & Glaser, 2021; Welch et al., 2010; Yang et al., 2018).

The increase in the prevalence of ASD (Maenner et al., 2023), the fact that these individuals are typically enthusiastic about using digital media technology (Chen et al., 2019; Lahiri et al., 2013; Moore & Calvert, 2000; Parsons & Mitchell, 2002; Schmidt & Schmidt, 2008), and have strong visual memories (Bozgeyikli et al., 2018) have led to the view that digitally delivered interventions may be beneficial for individuals with autism. The aforementioned factors, in light of the technological advancements, conventional methodologies have been supplanted by digital approaches in the education of children with ASD (Mazon et al., 2019). Technology-assisted instruction and intervention (TAII) represents an evidence-based practice for individuals with ASD (Hume et al., 2021). These interventions encompass the use of more common technologies, such as computers and mobile devices, as well as more advanced technologies, including virtual reality (VR), augmented reality (AR), mixed reality (MR), and robots (Carnett et al., 2023).

Coates (1992) defined VR as electronic simulations that can interact with three-dimensional environments through head-mounted displays and wired clothing. Gold (1993) defined it as an alternative world full of computer-generated images that can react to human movements, supported by expensive hardware such as video and data gloves. However, advances in display technologies have rendered the costly and scarce devices referenced in the definitions accessible to the general public (Srivastava et al., 2019). In virtual reality (VR) systems, stimuli are presented to users in three dimensions. This is frequently achieved through the use of a computer monitor, head-mounted display (HMD), and CAVE (Cave Automatic Virtual Environment). Desktop VR, also called non-immersive VR, is the simplest form of VR, presenting users with high-resolution panoramic images via a standard desktop computer. In desktop VR, users use a mouse or keyboard to move and explore the virtual environment (Berki, 2019). HMDs are a type of virtual reality (VR) headset that provides the user with a fully immersive virtual environment. CAVE is a system that presents a three-dimensional environment using two-dimensional projected images arranged around the user, thereby creating an immersive environment. In comparison to CAVE, HMD VR and desktop VR are more convenient and provide users with more cost-effective solutions (Feng et al., 2022). The appeal of VR environments, coupled with the ability of users to interact with and control virtual objects and focus on the environment with ease, has positioned this technology as an effective tool in educational processes (Chen, 2010; Wang et al., 2022). As virtual reality technology is capable of creating a three-dimensional simulation of real-world environments (Baileson et al., 2008) and allows for the repetition, manipulation, and control of social situations (Lorenzo et al., 2016; Parsons, 2016; Siano et al., 2015), its use in the field of autism spectrum disorder (ASD) is inevitable. Given that social interaction and communication disorders are the most prevalent issues among children with ASD (APA, 2022), VR interventions have been primarily directed towards social skills training (Lorenzo et al., 2016; Yiğit & Sani-Bozkurt, 2021).

In order to gain an understanding of the existing literature on the use of virtual reality in the context of autism spectrum disorder (ASD), we undertook an initial review of previous studies in this field. In a systematic review study, Mesa-Gresa et al. (2018) sought to elucidate the effects of virtual reality on ASD. The researchers did not impose any limitations with respect to the skills under consideration and included a total of 51 articles in their research. Mesa-Gresa et al. (2018) demonstrated that the area of social skills was the most extensively studied. In their review study, Thai and Nathan-Roberts (2018) concentrated on the role of social skills in virtual reality (VR) use. The study comprised a mere five pages and encompasses only nine articles. In their systematic review, Li et al. (2023) synthesized virtual reality studies on individuals with ASD conducted between 2010 and 2022, revealing that the most prevalent area of focus was social and emotional skills. Similarly, Yiğit and Sani-Bozkurt (2021) obtained comparable findings. The fact that social skills are the area of focus for the majority of researchers in this field demonstrates the necessity for further investigation. Liu (2023) and Kadir et al. (2023) conducted reviews of VR applications with a focus on social skills in children with ASD. However, only five studies were included in both reviews. In recent years, there has been a surge in more affordable and user-friendly VR technology, expanding its accessibility beyond specialized research settings. For instance, head-mounted displays have become increasingly cost-effective, and desktop-based systems now require minimal additional equipment. These advances indicate that earlier reviews may not fully capture the latest technological possibilities, thereby underscoring the need for a new comprehensive analysis of VR-based interventions. Unlike previous reviews, this systematic review examined 31 studies published over the past decade (2014–2024), focusing exclusively on children with ASD. This approach offers a more up-to-date and comprehensive perspective on how VR technology contributes to social skill development in childhood. In addition, different VR systems—such as CAVE, desktop VR, and HMD—were compared in terms of cost, technical requirements, and multi-user support, providing a more nuanced assessment of their impact on social skills. By also addressing topics rarely covered in earlier reviews, such as age restrictions, cybersickness, and gender balance, the methodological scope of the study is broadened. Despite limitations related to small sample sizes and issues with generalization, this review highlights the strong potential of VR-based interventions to enhance social skills in children with ASD and aims to fill the gaps noted in the existing literature. The lack of a recent comprehensive systematic review in this regard increases the importance of this study.

The objective of this systematic review is to provide a comprehensive summary of studies employing virtual reality (VR) interventions to enhance social skills in individuals with autism spectrum disorder (ASD) over the past decade. This review will evaluate the literature, including the methodology utilized, the targeted social skills, the VR technology employed, and will facilitate comparison of different studies in terms of their methods and execution procedures for those engaged in research in this field. Therefore, to guide this review and provide a clear focus, we formulated the following research questions:

- RQ1: Which types of VR interventions (e.g., CAVE, desktop VR, HMD) have been utilized in teaching social skills to children with ASD between 2014 and 2024?
- RQ2: Which specific social skill domains (e.g., emotion recognition, social interaction, nonverbal communication) are most commonly targeted?
- RQ3: What methodological approaches (e.g., experimental, quasi-experimental, single-subject, mixed method) are used, and how do they influence the reported outcomes?
- RQ4: What are the primary advantages and limitations (e.g., cost, cybersickness, age constraints, generalization issues) in using VR to support social skill development in ASD?

## Method

### Search Procedure

In the context of the systematic review, three researchers conducted a simultaneous search of the Google Scholar, EBSCOhost, IEEE Xplore, ScienceDirect, and PubMed databases. The assistance of Boolean operators (AND, OR, NOR, or AND NOT) was utilized to enter different combinations of keywords, which were then read and evaluated based on previously determined criteria. The study was limited to studies conducted between 2014 and 2024 to ensure the inclusion of studies conducted in the last 10 years. Furthermore, studies published in a peer-reviewed journal and in English were prioritized. The scans were recorded using the program Covidence (Covidence, 2024). The last search in the databases was conducted in April 2024. The keywords and search strategy are provided in Table 1.

**Table 1:** Search strategy

Population		Intervention		Outcome
Autism	**AND**	Virtual Reality	**AND**	Social Skill
**OR**		**OR**		**OR**
Autism Spectrum Disorder		VR		Interpersonal Skill
**OR**		**OR**		**OR**
ASD		Virtual Experience		Social Communication
**OR**				
Autistic				

### Inclusion and Exclusion Criteria for Studies

Inclusion criteria during the screening process were as follows:

1. Peer-reviewed studies published in English within the last 10 years (2014–2024).
2. Studies where the main intervention group comprises children with ASD (additional participants with typical development or other diagnoses may be included for comparison).
3. Empirical studies.
4. Studies focusing on social skills interventions.
5. Studies involving virtual reality (VR) as a form of human–computer interaction.

Exclusion criteria were as follows:

1. Grey literature (e.g., theses, posters).
2. Non-empirical studies.
3. Studies published before 2014.
4. Studies not written in English.
5. Studies that do not primarily target ASD participants (e.g., fewer than half the participants have ASD, or the intervention was not designed for ASD-specific needs).

### Data Extraction

After applying the inclusion and exclusion criteria, the authors of this study obtained the following data from the 31 included studies: year of the study, setting in which the study was conducted, diagnoses of the participants, age range and gender of the participants, social skills addressed in the study, main technology used for both hardware and software, and methodology and results.

## Results

A total of 634 studies were identified through a comprehensive search of the following databases: Google Scholar (n = 357), EBSCOhost (n = 211), IEEE Xplore (n = 25), ScienceDirect (n = 21), and PubMed (n = 20). Furthermore, the initial researcher conducted an additional search on artificial intelligence but was unable to identify any additional studies apart from those already identified. Additionally, two further studies were accessed through the bibliographies of the identified studies. Following the removal of duplicates (n = 336), the remaining 300 studies were then scanned independently and simultaneously by all three authors, with irrelevant studies (n = 199) subsequently eliminated.

**Fig. 1:**
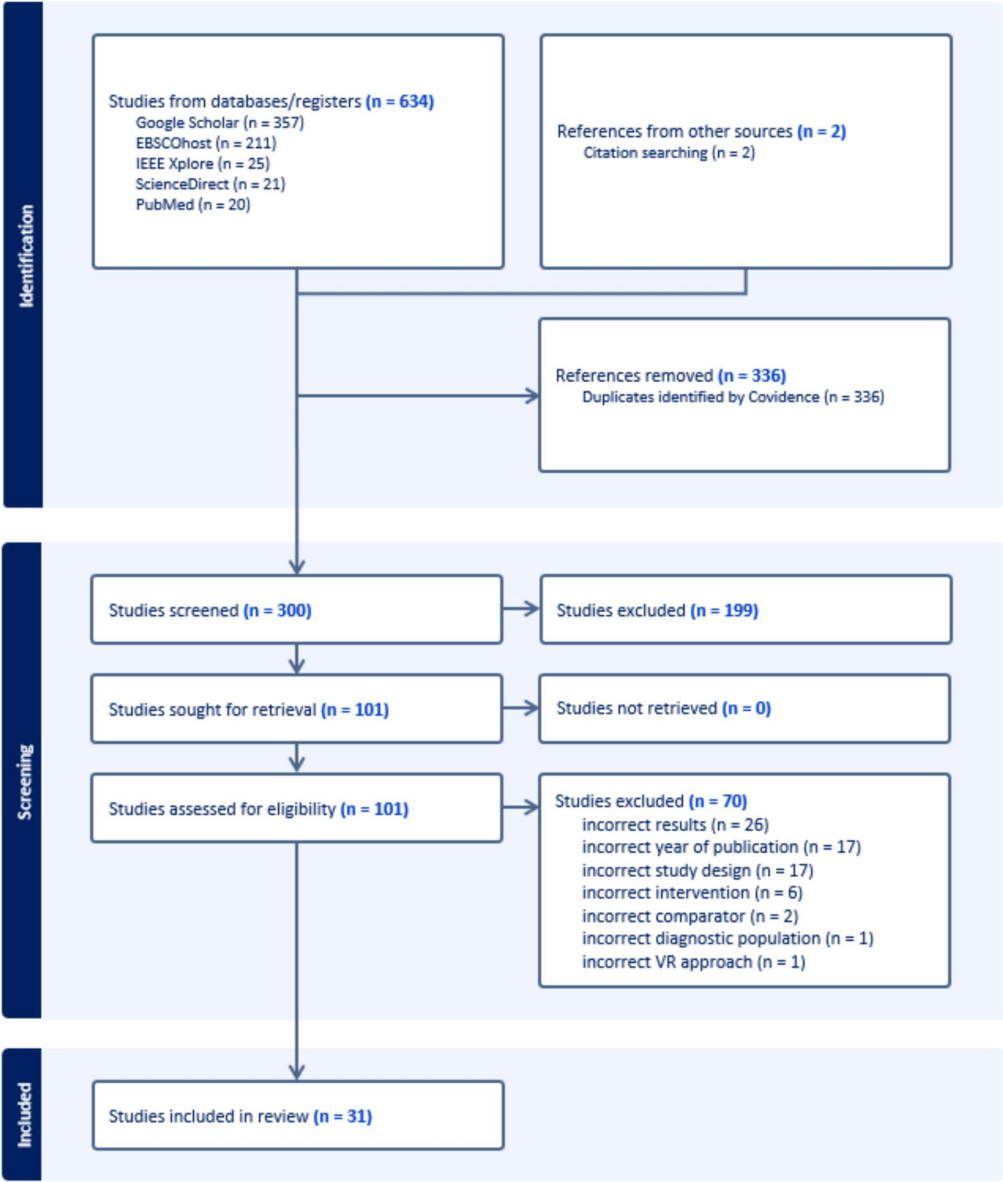
Flow chart

We examined the full texts of 101 studies meeting our initial inclusion criteria. After resolving any discrepancies among the authors, we excluded 70 studies from further consideration. The reasons for exclusion were as follows: 26 studies were excluded due to incorrect results, 17 due to incorrect year of publication, 17 due to incorrect study design, 6 due to incorrect intervention, 2 due to incorrect comparator, 1 due to incorrect diagnostic population, and 1 due to incorrect VR approach. Incorrect study design referred to studies that were non-empirical or lacked an appropriate methodological framework (e.g., purely theoretical papers, commentaries). Incorrect comparator indicated that the control or comparison intervention did not align with our VR focus (e.g., comparing two non-VR methods). Incorrect results pertained to studies that did not actually measure or report outcomes related to social skills, or whose reported results were insufficiently detailed to assess effectiveness. Finally, incorrect VR approach referred to studies utilizing a mode of VR application outside our definition (e.g., a non-HMD or non-desktop approach, or a different technology that did not meet our VR criteria). The remaining 31 studies were included in the study, and data extraction was conducted. The data obtained as a result of the data extraction process are analyzed below under the following headings: diagnoses of the participants; age range and gender of the participants; social skills addressed in the study; main technology used for both hardware and software; methodology; limitations and improvements in the study; and conclusions. The data flow chart is provided in Fig. 1 for reference (Table 2).

**Table 2:** Included studies

Author	Diagnosis	Participants	Research Method	Aim	VR Type	Instruments	Intervention Format	Limitations	Results
Cheng et al., 2015	ASD	EG: 3 (3M) CG: - Age: 10–13	Single-subject design	Social understanding and social interaction non-verbal communication	HMD	HMD (Model: I-Glasses PC 3D Pro), Laptop (a Pentium 3650MHZ processor and 64 MB RAM Windows XP). 3DMax and Poser software	Individual format; participants practiced social cues and non-verbal communication through immersive HMD scenarios	Whether participants continued to demonstrate social understanding, social interaction, and nonverbal communication in daily life was not evaluated	Improved social understanding and skills post-VR; beneficial for these individuals
Beach & Wendt, 2016	ASD	EG: 2 (2M) CG: - Age: 8–15	Case Study	Social interaction	HMD	Second Life HMD Oculus rift	Individual scenario practice; limited number of scenarios	The study only included two participants, limiting the generalizability of the results. The researchers only had three scenarios for each participant to practice	In the scenarios presented via VR, both participants were able to show gains in their weak areas. The first participant was able to continue the conversation without directing it to his or her particular interests. The other participant-maintained eye contact in most of the scenarios
Didehbani et al., 2016	ASD + ADHD	EG: 30 (26M, 4F) CG: - Age: 7–16	Case Study	Emotion recognition, social attribution	Desktop	Second Life 2.1, Microsoft Windows XP, 1.5 GHz 86 CPU using a 24-inch monitor with a resolution of 1920 – 1200 and MorphVox	Avatar-based role-play for emotion recognition; participants interacted in desktop virtual environments	Relatively small sample size and VR technology's lack of ability to display facial emotions on avatar faces in real time	Notable improvements in emotion recognition, social attribution, and executive function
Ip et al., 2016	ASD	EG: 52 CG: - Age: 6–11	Experimental	Emotion recognition, affective expression and social reciprocity	CAVE	CAVE-like immersive VR environment, Xbox 360 game controller	Immersive, CAVE-based scenarios focusing on emotional cues and reciprocity		Significant pre-post differences in emotion recognition, expression, and social reciprocity
Ke & Lee, 2016	LEVEL 1 ASD + TD	EG: 3 (1M, 2F) CG: - Age: 8–11	Mixed Method	Social flexibility, identity construction and norm construction	Desktop	OpenSimulator-based VR	Collaborative design in a desktop VR environment; children built and explored virtual worlds to practice flexibility	The project focused solely on three VR-mediated social interaction competencies and focused solely on the experience and performance of children with Level 1 ASD	VR-based collaborative design enhanced flexibility, identity, and norm construction skills in Level 1 ASD children
Lorenzo et al., 2016	ASD	EG: 20 (14M, 6F) CG: 20 (15M, 5F) Age: 7–12	Mixed Method	Emotional competence	CAVE	Semi-Cave IVRSystem, 7 DoF Mitsubishi PA-10 Robot Arm, PHOTONFOCUS MV-D752-160-CL-8 camera, 3ds Max, PeopleMaker	Immersive environment for emotional behavior practice vs. desktop environment		Children showed emotional behavior improvements in real-school settings. More appropriate emotional behaviors in immersive VR vs. desktop VR
Adjorlu et al., 2018	ASD	EG: 5 (5M) CG: - Age: 9–11	Mixed Method	Sharing, turn taking and theory of mind	HMD	Autodesk Maya and Unity, HTC Vive, Computer (Intel i7 7700k processor, GTX 1080 GPU, and 16GB of DDR4 ram)	HMD-based role-play with turn-taking tasks; built on social stories and video models	Due to technical errors, teachers could not communicate with students via microphone	HMD-based VR can teach social skills (sharing, turn-taking) in combination with social stories and video modeling advantages
Babu et al., 2017	LEVEL 1 ASD + TD	LEVEL 1 ASD: 8 (4M, 4F) TD: 8 (7M, 1F) Age: 14–15	Experimental	Social interaction	Desktop	Task computer (17″ monitor), number keypad, eye-tracker goggles	Eye-tracking-based training to improve social gaze and interaction in a desktop VR	The use of a wearable eye tracker with a chinrest and keypad is not suitable for low-functioning autism Small sample size	VR-based system can affect gaze-related indices, potentially indicating anxiety levels and improving social gaze in Level 1 ASD participants
Ip et al., 2018	ASD	EG: 36 (31M, 5F) CG: 36 (33M, 3F) Age: 7–10	Experimental	Enhancing emotional and social adaptation skills	CAVE	CAVE VR system. Intel Xeon Processor E5-2687W, NVIDIA Quadro M6000 graphics cards, NVIDIA Sync card, 128GB of system RAM, OptiTrack Flex, Unity 3D	Immersive environment focusing on emotion expression, regulation, and social interaction		Strong in-class improvement in emotion expression/regulation, but no significant gains in emotion recognition measures
Ke & Moon, 2018	LEVEL 1 ASD	EG: 8 (7M, 1F) CG: - Age: 10–14	Mixed Method	Social interaction, collaboration game	Desktop	Opensimulator-based VR	Collaborative gaming in a desktop VR setting. Participants engaged in group tasks to practice social collaboration	Study involves a relatively small sample and mainly in-game performance measures, due to its focus on in-situ, design-based research of VR gaming for autistic learners	Collaborative VR gaming enhanced social interaction practice and performance in Level 1 ASD children
Zhang et al., 2018	ASD + TD	ASD: 7 (6M, 1F) TD1: 7 (6M, 1F) TD2: 14 (12M, 2F) Age: 7–17	Experimental	Collaborative interaction and communication skills	Desktop	Two Dell desktop computers T3610 (E5-220 V3 CPU and 8GB RAM)	Multi-user desktop VR environment (CoMove). Participants practiced collaborative tasks, monitored communication	Only one coder coded participants' communication behaviors, no generalizations were made, and finding ways to enable and encourage face-to-face communication is a challenge for all CVEs	Children with ASD showed improvements in collaborative measures after using the CoMove system
Zhao et al., 2018	ASD + TD	ASD: 12 TD: 12 Age: 12–13	Experimental	Social interaction, collaboration game	Desktop	HIH CVE system, Leap Motion, a set of headset and webcam, Tobii EyeX tracker	Cooperative gaming in a desktop VR environment. Participants played partner-based games		Participants enjoyed the cooperative games, improved collaboration, and recognized importance of communication to succeed
Herrero & Lorenzo, 2020	ASD	EG: 7 (6M, 1F) CG: 7 Age: 8–15	Case Control Study	Social and emotional reciprocity, Non-verbal communication	HMD	HDM Oculus Rift, Unity 3, Smartphone with Android OS, tripod	HMD-based sessions to practice social reciprocity and nonverbal cues	The lack of analyze the durability of improvements in the long term, duration of the intervention, and heterogeneity of participants prevented generalization of the results	As a result of the research, improvements were seen in all areas such as Social and emotional reciprocity, non-verbal communication, flexibility to changes, stereotypes and sensorial reactivity in the participants in the experimental group
Ke et al., 2022a	ASD	EG: 7 (6M, 1F) CG: - Age: 10–14	Multiple single-case study	Initiating social interactions. interpersonal negotiation self-identity expression and flexible thinking	Desktop	Desktop VR	Multiple single-case approach: participants practiced initiating interactions via desktop VR tasks	Small sample size	Positive and significant improvement from baseline to intervention phases in social skills performance
Zhang et al., 2020	ASD + TD	EG: 20 (16M, 4F) CG: 20 (16M, 4F) Age: 13.50 avg	Experimental	Social communication and collaboration	Desktop	Desktop VR	Intelligent agent in desktop VR to detect/evaluate social communication	Small sample size, small number of system-generated features	The agent accurately assessed social communication/cooperation, showing promise for independent evaluation in ASD
Tsai et al., 2020	ASD	EG: 3 (3M) CG: - Age: 7–9	Multiple single-case design	Emotion recognition	CAVE	CAVE VR system, Microsoft Kinect, projector (K300ST)	Role-playing performance in a CAVE system to see and imitate facial expressions and body language	CAVE-like systems are difficult to establish in schools, Perspective Confusion, more focus on body movements, individual differences	An increase in role-playing performance was observed in all three participants. The system is effective on children with ASD seeing, understanding and imitating facial expressions and body language
Amat et al., 2021	ASD + TD	ASD: 9 (5M, 4F) TD: 9 (5M, 4F) Age: 10–13	Experimental	Joint attention, gaze tracking	Desktop	Interactive Virtual Reality System, e Tobii EyeX, Autodesk Maya	Gaze-based VR tasks to improve joint attention. Participants used eye-tracking in desktop VR scenarios	Small sample size, Failure to transfer social skills to daily life, no control group	Participants with ASD showed significant improvement in their performance in all speed groups. This statistically significant improvement indicated that InViRS was able to help children with ASD to adapt and respond to the changes in gaze prompts speed
Moon & Ke, 2019	LEVEL 1 ASD	EG: 15 (13M, 2F) CG: - Age: 10–14	Mixed Method	Responding, initiating an interaction, negotiation, and collaboration	Desktop	Opensimulator-based VR	VR-based social skills training with scenario-based practice for HFASD individuals	Small sample size	VR-based social skills training has been helpful in social skills training for children with LEVEL 1 ASD. Additionally, this study demonstrated that social scenario types mediate treatment integrity of VR-based social skills training
Elkin et al., 2022	ASD	EG: 10 CG: - Age: 12.53 avg	Case Study	Gaze Fixations, Visual Searching	HMD	HTC Vive Pro Eye VR headset, Area 51 laptop computer, Tobii eyeX	Eye-tracking in an HMD environment to analyze gaze fixations on virtual avatars	Small sample size. In study utilized only one measurement of gaze and visual searching behavior	The study found that persons with moderate ASD had 4 to 19 times greater gaze fixation on the virtual avatar than those with mild ASD with various facial regions. Individuals with moderate ASD were generally found to have a higher tendency to shift their gaze to the floor than participants with mild ASD after making eye contact with various locations on the avatar's face
Frolli et al., 2022	LEVEL 1 ASD	EG: 30 (25M, 5F) CG: 30 (26M, 4F) Age: 9–10	Experimental	Emotion recognition	Desktop	Desktop VR, 3D projection, Emotions photos	Desktop-based VR tasks for primary emotion recognition	The study specifically focused on children with LEVEL 1 ASD; The sample included mainly male participants	Acquisition times for primary emotion recognition were identical for both VR and traditional methods; secondary emotion tasks improved faster with VR
Ip et al., 2022	ASD	EG: 48 (41M, 7F) CG: 59 (47M, 12F) Age: 6–12	Quasi-experimental design	Affective expression, social reciprocity	HMD	HMD OculusRift, Lenovo Legion Y520 laptop (NVIDIA GeForce GTX 1060 mobile graphics processing unit (GPU), 16GB of main memory, 256GB of a solid-state drive)	Educational VR program in HMD for affective expression, reciprocity in mainstream schools	Use of quasi-experimental design, the research team was unable to compare VR training to non-VR approaches with similar instructional content. No follow-up data available to examine the retention effect of the intervention. Gender imbalance	Improved emotional expression and social reciprocity in ASD children; no direct non-VR comparison but indicates feasibility
Ke et al., 2022b	ASD	EG: 4 (4M) CG: - Age: 9–11	Mixed Method	Social Interaction, Self-expression, Cognitive flexibility	Desktop	Opensimulator-based VR	Web VR-based informal learning with collaborative/social tasks	Small sample size, All participants were male	The current study findings provide preliminary evidence that a web VR-based informal learning program is feasible and provides a positive impact on the practice and development of complex social skills for children with autism
Meng & Yeh, 2022	ASD	EG: 10 CG: - Age: 7–12	Mixed Method	Social Interaction	HMD	Desktop computer, HTC VIVE HMD, headset microphone, camera	HMD-based social interactions. Students practiced dialogues and environmental adaptation	Small sample size	Effectively improved social skills in school life, environmental adaptation, and attempts to initiate dialogue
van Pelt et al., 2022	ASD	EG: 26 (21M, 5F) CG: - Age: 18–62	Experimental (uncontrolled single group)	Emotion perception, Social Perception, Theory of Mind, Social interaction	HMD	HMD OculusRift, Microsoft Xbox One joystick, MorphVox	HMD-based tasks focusing on social cognition, sensitivity, facial affect perception	Uncontrolled pilot study, small sample size	Participants and therapists found the intervention acceptable; increases in social cognition/sensitivity, decreases in social anxiety
Zhao et al., 2022	ASD	EG: 22 (19M, 3F) CG: 22 (16M, 6F) Age: 3–5	Experimental	Social interaction, Emotional expression	HMD	HMD-based VR, Computer	Short-term intervention (3 months). HMD environment for cognitive, imitation, social interaction improvement	Small sample size. There was only a 3-month intervention in the study. Nurses could not enter the virtual environment to offer clues to children	After intervention, the developmental abilities of both groups of children in the areas of cognition, imitation, and social interaction were improved over their abilities measured before the intervention
Kourtesis et al., 2023	ASD	EG: 25 (19M, 6F) CG: - Age: 19–52	Experimental	Social interaction, cognitive functioning	HMD	VRESS, HTC Vive Pro Eye headset	Immersive VR social skills training for adults with ASD	The high age of the participants. Lack of a randomized controlled trial (RCT)	The study provides significant evidence that implementing immersive VR social skills training in ASD is feasible, comfortable to use, and acceptable to adults with ASD
Manju et al., 2023	ASD	EG: 10 CG: - Age: 8–12	Mixed Method	social communication, eye tracking	Desktop	Desktop VR, Unity Software	Desktop-based VR. Compared to three traditional interventions (DTT, ABA, NDBI)		The VR system proposed in the study showed better results compared to three other traditional interventions such as Discrete Trial Training (DTT), Applied Behavior Analysis (ABA), and Natural Developmental Behavioral Interventions (NDBI)
Moon & Ke, 2023	ASD	EG: 4 (2M, 2F) CG: - Age: 12–13	Single-case experimental design	Social interaction, Self-identity	Desktop	Opensimulator-based VR	Adaptive prompts vs. non-adaptive prompts in a desktop VR scenario	The speech data mining in this study was limited to gathering data on the emotional states of autistic children. The current study’s data collection approach was limited in that it only gathered voice data from the study participants. Small sample size	It was observed that during VR-based training, participants' positive social skill performances showed mixed results, while their negative social skill performances decreased. Using adaptive prompts increased the performance of some social skills (i.e., social interaction initiation and interpersonal negotiation) more than using non-adaptive prompts
Soltiyeva et al., 2023	ASD	EG: 12 (8M, 4F) CG: - Age: 4–15	Experimental	Social interaction, Communicational skills	HMD	VR Oculus Quest2, computer	HMD-based sessions to explore virtual character interaction. Participants freely navigated VR worlds	The small number of participants and their different ages and different autism levels	More than 8 children were active and tried to interact with a virtual character. They focused on details/static objects
Kim et al., 2024	ASD	EG: 14 (12M, 2F) CG: - Age: 16–34	Mixed Method	Workplace social skills and self-efficacy	HMD	WorkplaceVR, Unity3D, Windows 10 (Microsoft) PC (Intel Core i7, GeForce RTX 2070 graphics card, and 16 GB RAM), VIVE Pro Eye VR headset (HTC), Empatica E4 wristband	Workplace role-play in HMD VR to build job-related social skills	Only participants with autism who could communicate and interact with others were included in the study. Failure to transfer social skills to daily life	VR system significantly improved self-efficacy in workplace scenarios for participants with autism
Moon, 2024	ASD	EG: 4 (2M, 2F) CG: - Age: 12–13	Case Study	Verbal prompts, Social Interactions	Desktop	Opensimulator-based VR	Verbal prompt-based VR sessions for social interaction improvement	Small sample size, Failure to transfer social skills to daily life	VR-based education contributed to academic understanding of verbal prompts for social behaviors. Students responded quickly and accurately to social cues in various VR simulations

### Participants’ Diagnoses

As the diagnostic group was defined as ASD in the inclusion criteria of the study, the studies included children diagnosed with ASD. A total of 31 studies were included in the review, comprising a total sample size of 710 participants. Of these, five studies (Babu et al., 2017; Frolli et al., 2022; Ke & Lee, 2016; Ke & Moon, 2018; Moon & Ke, 2019) specifically focused on children with Level 1 ASD. Although some of the studies we reviewed use the term *‘High-functioning autism (HFASD)’*, the literature points to ongoing debates surrounding this label (Alvares et al., 2020; Bölte, 2023; Eldridge et al., 2025). In particular, describing individuals as ‘high-functioning’ can underestimate their diverse support needs. Therefore, to align with more contemporary and inclusive terminology, we have chosen to adopt ‘*Level 1 ASD*,’ referring to a DSM-5 classification for individuals requiring relatively lower levels of support. This approach maintains fidelity to the original studies’ terminology while acknowledging evolving perspectives on autism and the nuances of support requirements. It is noteworthy that the study by Didehbani et al. (2016) included children with Attention Deficit and Hyperactivity Disorder (ADHD) who also had ASD. Twenty-five studies included interventions exclusively for children with ASD, while six studies (Amat et al., 2021; Babu et al., 2017; Ke & Lee, 2016; Zhang et al., 2018, 2020; Zhao et al., 2018) also included children with typical development. The distribution of participants according to their diagnoses is illustrated in Table 3.

**Table 3:** Diagnosis, Age Group, and Gender Distribution (N = 710)

	n	%
Diagnosis		
ASD	533	75.1
Typical Development (TD)	71	10.0
Level 1 ASD	93	13.1
ASD + ADHD	13	1.8
Total	710	100.0
Age Group		
0–6 years	44	6.2
6–15 years	599	84.4
15 + years	67	9.4
Total	710	100.0
Gender		
Male	485	68.3
Female	113	15.9
Unknown	112	15.8
Total	710	100.0

### Demographic (Age and Gender) Distribution of Participants

A review of the data extraction studies revealed that the age range of the participants was between 3 and 62 years. Companies that offer virtual reality (VR) applications, such as Oculus Rift and Playstation, have stated that this technology should not be used by individuals under the age of 12 or 13. Additionally, View-Master VR, a VR equipment developed exclusively for children, has specified this age as 7 for its own equipment (Chen et al., 2019). The fact that only one study (Zhao et al., 2022) among the studies included in this systematic review had participants under the age of 6 lends further support to this information.

A total of 31 studies included in the systematic review provided data on the age of participants, which was reported in a variety of categories. In some studies, only the mean age was reported, which made it challenging to conduct a comprehensive analysis of the age distribution of the participants. Moreover, the age ranges of the participants in the studies exhibited considerable variation. The aforementioned diversity renders a comprehensive evaluation of age groups challenging. For this reason, in our study, we employed a more granular classification of age ranges, delineating three broad categories: 1–6 years, 6–15 years, and 15 + years, in order to facilitate a more meaningful and consistent analysis of the data. This approach allows for more robust age-based analyses and a more general interpretation of the results. A total of 1 study included participants aged 1–6 years (Zhao et al., 2022), while 26 studies included participants aged 6–15 years (Adjorlu et al., 2018; Amat et al., 2021; Babu et al., 2017; Cheng et al., 2015; Didehbani et al., 2016; Elkin et al., 2022; Frolli et al., 2022; Herrero & Lorenzo, 2020; Ip et al., 2016, 2018, 2022; Ke & Lee, 2016; Ke & Moon, 2018; Ke et al., 2022b). The following studies were also considered: Ke et al. (2022b), Lorenzo et al. (2016), Manju et al. (2023), Meng and Yeh (2022), Moon and Ke (2019, 2023), and Moon (2024). Additionally, Soltiyeva et al. (2023) and Tsai et al. (2020) were included. A total of 20 studies were identified, of which 16 included participants aged 15 and above (Beach & Wendt, 2016; Kim et al., 2024; Kourtesis et al., 2023; van Pelt et al., 2022). The distribution of participants according to age groups is as follows: 44 participants between the ages of 1 and 6, 599 participants between the ages of 6 and 15, and 67 participants aged 15 and above. The distribution is presented in Table 3 for reference.

Upon analysis of the gender of the participants in the studies, it was observed that 5 studies (Elkin et al., 2022; Ip et al., 2016; Manju et al., 2023; Meng & Yeh, 2022; Zhao et al., 2018) lacked sufficient information regarding the gender of the participants. Upon analysis of the remaining 26 studies, it was determined that 485 of the participants were male and 113 were female. It is established that the prevalence of ASD is 43.0 among boys and 11.4 among girls (Maenner et al., 2023). Women are largely excluded from research studies (D’Mello et al., 2022), and due to the unique profiles of ASD in women (Hiller et al., 2016), there is a perceived deficiency in the representation of women in research. However, Burrows et al. (2022) proposed that the gender ratio can be balanced if diagnostic caveats are taken into account.

### Analysis of Social Skills

The studies included in this systematic review examine a range of social skills. It is crucial to identify which social skills are most frequently examined in studies in order to gain insight into current trends and gaps in the field. Rather than examining the impact of VR on a single social skill, many studies have chosen to assess its effects on multiple social skills. This demonstrates how VR technology can be used to develop a wide range of social skills, but also helps us to understand in which areas these skills are more effective. Consequently, an analysis of the social skills addressed in this systematic review will facilitate a more comprehensive understanding of the literature in this field.

A review of the studies revealed that 20 studies met the criteria for inclusion (Babu et al., 2017; Beach & Wendt, 2016; Cheng et al., 2015; Herrero & Lorenzo, 2020; Ip et al., 2016, 2022; Ke & Moon, 2018; Ke et al., 2022a, 2022b; Kim et al., 2024; Kourtesis et al., 2023; Manju et al., 2023; Meng & Yeh, 2022; Moon & Ke, 2023; Moon, 2024; Soltiyeva et al., 2023; van Pelt et al., 2022; Zhang et al., 2020; Zhao et al., 2018, Zhao et al., 2022). The studies included in this review were conducted by the following researchers: Babu et al. (2017), Beach and Wendt (2016), Cheng et al. (2015), Herrero and Lorenzo (2020), Ip et al., (2016, 2022), Ke and Moon (2018), Ke et al. (2022a, 2022b), Kim et al. (2024), Kourtesis et al. (2023), Manju et al. (2023), Meng and Yeh (2022), and Moon and Ke (2023). The studies examined social interaction and reciprocity. This figure demonstrates that the majority of studies concentrate on the subject of social interaction. Seven studies (Didehbani et al., 2016; Frolli et al., 2022; Ip et al., 2016, 2018; Lorenzo et al., 2016; Tsai et al., 2020; van Pelt et al., 2022) concentrated on the subject of emotion recognition and discrimination. Additionally, researchers have concentrated their efforts on developing skills such as joint attention (Amat et al., 2021; Elkin et al., 2022), cognitive and social flexibility (Ke & Lee, 2016; Ke et al., 2022a; Ke et al., 2022b), and negotiation (Ke et al., 2022a). Furthermore, studies have explored the role of joint attention (Amat et al., 2021; Moon & Ke, 2019, 2023), cooperation and cooperative play (Adjorlu et al., 2018; Ke & Moon, 2018; Zhang et al., 2018, 2020).

In order to provide a clearer understanding of how these interventions were implemented, we added an “Intervention Format” column to Table 2. This column highlights the specific methods used to teach social skills in each study, including avatar-based simulations (e.g., Cheng et al., 2015; Didehbani et al., 2016), multi-user collaborations (e.g., Zhang et al., 2018; Zhao et al., 2018), and role-play tasks supported by head-mounted displays (e.g., Adjorlu et al., 2018; Tsai et al., 2020). Notably, several studies (e.g., Herrero & Lorenzo, 2020; Lorenzo et al., 2016) employed immersive environments such as CAVE or HMD for more realistic social interactions, while others (e.g., Ke & Lee, 2016; Ke & Moon, 2018) focused on desktop-based collaboration, enabling multiple children to co-create or interact simultaneously. These diverse approaches indicate that no single ‘best’ method has emerged; rather, the choice of VR format appears to depend on resources, target social skills, and participant characteristics.

### Technology Utilized in Studies

**Fig. 2:**
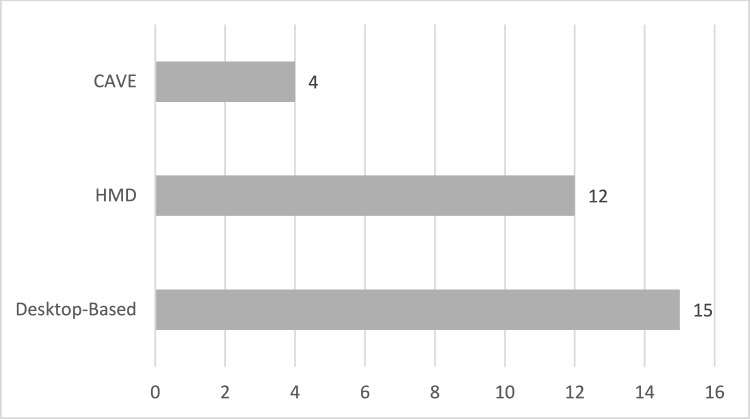
Technology utilized in studies

Virtual reality (VR) can be delivered using a variety of tools, including head-mounted displays (HMDs) and cave automatic virtual environments (CAVEs), as well as desktop and laptop computers. Virtual reality head-mounted displays (HMDs) are optical devices that provide the user with a fully immersive virtual environment. CAVE is a system that presents a three-dimensional environment using two-dimensional projected images arranged around the user, thereby creating an immersive environment. Virtual reality (VR) and CAVE systems afford users the ability to interact with the environment through the use of specialized controllers, computer vision techniques (such as motion capture), and other computer-based sensing systems (such as eye tracking and speech recognition). The virtual reality technology employed in the studies presented in this research can be classified into three categories: desktop-based, head-mounted display (HMD), and CAVE (Cave Automatic Virtual Environment). Fifteen studies were identified for review (Amat et al., 2021; Babu et al., 2017; Didehbani et al., 2016; Frolli et al., 2022; Ke & Lee, 2016; Ke & Moon, 2018; Ke et al., 2022a; Ke et al., 2022b; Manju et al., 2023; Moon, 2024; Moon & Ke, 2019, 2023; Zhang et al., 2018; Zhang et al., 2020; Zhao et al., 2018) employed a desktop VR system. HMD VR system was used in 12 studies (Adjorlu et al., 2018; Beach & Wendt, 2016; Cheng et al., 2015; Elkin et al., 2022; Herrero & Lorenzo, 2020; Ip et al., 2022; Kim et al., 2024; Kourtesis et al., 2023; Meng & Yeh, 2022; Soltiyeva et al., 2023; van Pelt et al., 2022; Zhao et al., 2022). In addition, four studies employed the CAVE VR system (Ip et al., 2016, 2018; Lorenzo et al., 2016; Tsai et al., 2020). Upon examination of the studies in their entirety, it becomes evident that a considerable variety of equipment materials has been employed. It is anticipated that this extensive range of equipment materials will facilitate future research endeavors. A detailed overview of the equipment used in the reviewed studies is provided in Table 2, while Fig. 2 illustrates the distribution of VR technologies—namely, CAVE, desktop, and HMD—employed in these interventions.”

### Methodological Approaches Utilized

**Fig. 3:**
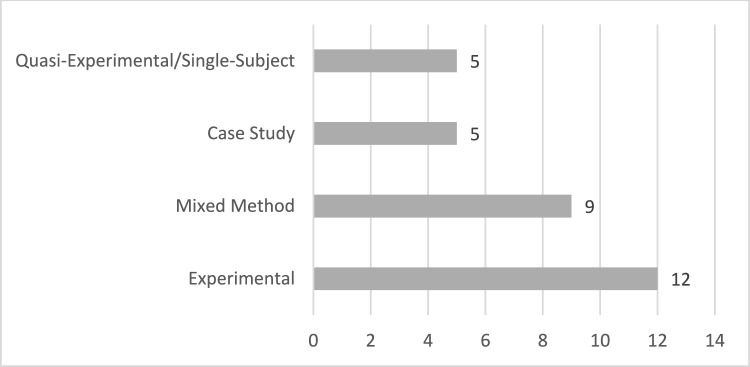
Methodological approaches utilized

Upon examination of the methodologies employed in the studies included in the research, it becomes evident that 12 studies (Amat et al., 2021; Babu et al., 2017; Frolli et al., 2022; Ip et al., 2016, 2018; Kourtesis et al., 2023; Soltiyeva et al., 2023) utilized an experimental approach. The mixed method approach was used in nine studies (Adjorlu et al., 2018; Ke & Lee, 2016; Ke & Moon, 2018; Ke et al., 2022b; Kim et al., 2024; Lorenzo et al., 2016; Manju et al., 2023; Meng & Yeh, 2022; Moon & Ke, 2019), five studies were case studies (Beach & Wendt, 2016; Didehbani et al., 2016; Elkin et al., 2022; Herrero & Lorenzo, 2020; Moon, 2024) and 5 studies employed a single-subject/quasi-experimental design (Cheng et al., 2015; Ip et al., 2022; Ke et al., 2022a; Moon & Ke, 2023; Tsai et al., 2020). The distribution of research methods employed is presented in Fig. 3.

### Identified Improvements and Limitations

The studies reviewed highlight the positive impact of VR-based interventions on children with ASD. For instance, Manju et al. (2023) found that their VR program surpassed established approaches—such as Discrete Trials Instruction (DTI), Applied Behavior Analysis (ABA), and Naturalistic Developmental Behavioral Interventions (NDBI)—in enhancing social skills. Other research reported meaningful gains in emotion recognition, collaboration, and turn-taking (Adjorlu et al., 2018; Amat et al., 2021; Zhang et al., 2018). Certain immersive systems (e.g., CAVE, HMD) appeared particularly effective in eliciting realistic social interactions or emotional expressions (Herrero & Lorenzo, 2020; Lorenzo et al., 2016), whereas desktop VR was often favored for its accessibility and ease of use (Ke & Lee, 2016; Ke et al., 2022b). Nevertheless, some studies observed mixed or limited effects: for example, Frolli et al. (2022) found no difference in the time required to recognize primary emotions between VR and traditional methods, and Ip et al. (2018) failed to detect significant improvements in emotion recognition.

Despite these advances, the literature also highlights several limitations. Chief among them are small sample sizes, which reduce statistical power and hinder generalizability (Didehbani et al., 2016; Babu et al., 2017; Beach & Wendt, 2016; Ke & Moon, 2018; Ke et al., 2022a; Zhang et al., 2020; Amat et al., 2021; Moon & Ke, 2019; Elkin et al., 2022; Ke et al., 2022b; Meng & Yeh, 2022; van Pelt et al., 2022; Zhao et al., 2022; Moon & Ke, 2023; Soltiyeva et al., 2023; Moon, 2024). Moreover, many designs lacked control groups, complicating direct comparisons between VR and non-VR interventions. Problems with generalization and retention of skills are another recurring concern (Amat et al., 2021; Beach & Wendt, 2016; Herrero & Lorenzo, 2020), given that improvements observed in virtual settings do not always translate seamlessly to everyday contexts. While only one study (Adjorlu et al., 2018) reported notable technical difficulties (e.g., faulty equipment), cost and setup complexity—particularly for fully immersive hardware—remain practical barriers (Tsai et al., 2020). Finally, many interventions were short-term and lacked follow-up data, limiting insight into the long-term sustainability of any gains (Zhao et al., 2022).

In summary, although VR appears promising for fostering social reciprocity, emotion recognition, and collaborative play in children with ASD, these findings are tempered by methodological constraints—especially small samples, brief intervention periods, and a lack of robust controls. Further research featuring larger, well-controlled trials and extended follow-up is needed to confirm whether the observed benefits persist beyond initial sessions and can be generalized to real-world settings.

#### .

## Discussion

In this systematic review, we examined 31 studies published over the past decade (2014–2024) that investigated the effectiveness of virtual reality (VR)-based social skills interventions for children with autism spectrum disorder (ASD). The results indicate that the majority of studies were published in 2018 (n = 6) and 2022 (n = 8), suggesting a growing interest in VR research in recent years. The findings further reveal that most studies focused on children aged 6–15 (n = 599), while there was limited participation from those under 3 years or over 15 years of age. Companies providing VR applications have recommended that VR technology not be used by individuals under the ages of 12 or 13 (Chen et al., 2019). For instance, the minimum age is specified as 10 for the Meta Quest (Meta, 2024), and 13 for the Apple Vision Pro (Apple, 2024). View-Master VR, developed exclusively for children, sets the lowest usage age at 7. These age-related restrictions, combined with the complexities of diagnosis and intervention processes in early childhood, may explain why research predominantly involves the 6–15 age group. Meanwhile, older adolescents and adults with ASD have different educational and therapeutic needs but appear to be underrepresented in VR studies, underscoring the importance of adapting both equipment and content for different age groups in future research.

A notable gender disparity was observed across the studies, with 485 male participants compared to 113 female participants. In addition to the established finding that ASD is more frequently diagnosed in males (Maenner et al., 2023), some evidence suggests that girls may be overlooked in the diagnostic process due to “camouflaging” of symptoms (D’Mello et al., 2022; Hiller et al., 2016). Further research is therefore needed on how to tailor VR interventions to girls’ unique profiles. This gender imbalance could limit the generalizability of current findings and serve as a critical consideration for future research.

Social and communicative deficits in ASD can be grouped into three categories—social-emotional deficits, nonverbal communication deficits, and difficulties in establishing and maintaining relationships (APA, 2022). The studies included in this review addressed all three categories. Social interaction with virtual avatars or peers was the most prominently targeted skill area, wherein various VR-based scenarios were presented to participants, and their ability to engage in social interaction was subsequently assessed. Compared to other disability groups, individuals with ASD experience greater challenges in comprehending and responding to emotional expressions (Black et al., 2017). However, emotional recognition in children with ASD is generally inconsistent, partly due to substantial differences in sample sizes, tasks, and participant characteristics (Lievore et al., 2023). The studies reviewed here that involved emotion recognition and discrimination (Didehbani et al., 2016; Frolli et al., 2022; Ip et al., 2016, 2018; Lorenzo et al., 2016; Tsai et al., 2020; van Pelt et al., 2022) also exhibited variations in outcomes. For example, while participants in the studies by Didehbani et al. (2016), Tsai et al. (2020), and van Pelt et al. (2022) showed improvements in emotion recognition following VR-based interventions, Frolli et al. (2022) reported no difference in acquisition times for emotion recognition when comparing traditional methods and VR. Ip et al. (2016) found positive differences in pre- and post-test emotion recognition scores, whereas a subsequent study (Ip et al., 2018) reported no significant improvement. These discrepancies may stem from differences in participant profiles or the specific VR scenarios employed. Future research could address such inconsistencies by standardizing emotion recognition protocols, enhancing avatar realism, and examining diverse participant groups (e.g., level 1 ASD, those with comorbid diagnoses).

Among the various VR systems employed for ASD interventions—CAVE, desktop, and head-mounted display (HMD)—each offers distinct features. The choice of system is particularly important for practitioners. In this review, desktop-based systems emerged as the most frequently used, a finding consistent with Dechsling et al. (2021). However, a systematic review by Yiğit and Sani-Bozkurt (2021) reported desktop-based systems as the least used. While the studies included here suggest that various VR systems can enhance the social skills of individuals with ASD, the question of which system is most suitable for teaching social skills remains open. Lorenzo et al. (2016) found that immersive environments may elicit more appropriate emotional behaviors compared to desktop settings, and other studies (Chen et al., 2019; Herrero & Lorenzo, 2020) similarly support the utility of immersive VR systems. Yet, CAVE systems require more equipment than HMD or desktop VR, potentially escalating costs. Notably, only four of the studies (Ip et al., 2016, 2018; Lorenzo et al., 2016; Tsai et al., 2020) used CAVE, indicating limited comparative data across multiple VR formats. Additionally, Halabi et al. (2017) applied three VR types to both children with ASD and typically developing peers and found the highest satisfaction levels with CAVE systems, as well as shorter response times in children with ASD. Cooperative VR environments (supporting multiple users simultaneously) are also believed to be effective for social skills training.

Special education is a highly diverse field, requiring multiple research designs (Odom et al., 2005). Alongside the four frequently used approaches—descriptive, correlational, experimental, and qualitative—mixed-method studies have become increasingly popular (Cook & Cook, 2016). Methodologically, most studies in this review employed experimental or quasi-experimental designs, yet only nine included a control group (Amat et al., 2021; Kourtesis et al., 2023; van Pelt et al., 2022). The absence of control groups limits the capacity to conclusively compare VR interventions with alternative methods. Small sample sizes (n = 16 studies) and difficulties in generalization (n = 8 studies) were also commonly reported. The challenges that children with ASD face in generalizing skills (Yerys et al., 2009) are closely tied to how closely VR environments mimic real-life conditions (Rajendran, 2013). Hence, factors like high-resolution visuals, avatar realism, and scenarios reflective of daily life contexts could be critical for success in future studies.

Moreover, cybersickness was mentioned in only one study (Kourtesis et al., 2023), suggesting that this issue is often overlooked in VR research. This gap may indicate a lack of consistent protocols or measurement tools for safeguarding participant well-being. Symptoms such as nausea and dizziness could pose (Davis et al., 2014; Stauffert et al., 2020) particular challenges for children with ASD, who often have heightened sensory sensitivities. Although recent hardware and software aim to minimize these effects by reducing latency and jitter (Farmani & Teather, 2020), more sensitive measurement and reporting tools are needed to fully address cybersickness in VR studies.

In conclusion, the reviewed studies suggest that VR-based interventions can serve as a valuable tool for enhancing social skills in children with ASD. Nevertheless, several factors—small sample sizes, absence of control groups, gender imbalances, and cybersickness—limit the scope and robustness of current findings. Future research should incorporate more diverse samples in terms of age and gender, conduct comparative analyses of different VR systems, and employ controlled, longitudinal designs (including follow-up measurements) to yield more generalizable results. Such efforts will help clarify how VR technology can sustainably contribute to the social skills development of children with ASD.

## Conclusions

In conclusion, this study examined the effects of using virtual reality (VR) technology in social skills training by synthesizing the findings of studies conducted over the past decade, including VR interventions for social skills of individuals with autism spectrum disorder (ASD). The findings indicated that VR-based interventions can facilitate the creation of a secure environment in which individuals with ASD can engage in the practice of social skills in a manner that is both safer and more comfortable. Furthermore, these interventions have the potential to enhance participation by providing participants with tasks that are motivating and engaging. The use of VR technology is becoming increasingly commonplace, with new equipment being introduced on a daily basis. Nevertheless, it is imperative that experts exercise caution when utilizing VR technology, particularly in individuals with ASD. In light of the findings and cautions articulated by the research sponsors, it is evident that this technology is appropriate for individuals above the age of 12 or 13. All varieties of VR have exhibited promise in the field of social skills instruction. It is established that desktop-based systems are the most cost-effective option; however, users may prefer CAVE-style VR systems. Further research is required to elucidate the distinctions and consequences of the various types of VR, namely HMD, CAVE, and desktop-based systems. It is recommended that future studies address the limitations of existing research, including the use of small sample sizes and the lack of generalizability. Additionally, there is a need for more studies on gender balance in VR research. Overall, the evidence suggests that VR technology has the potential to enhance the social skills of individuals with ASD, but further research is necessary to confirm this hypothesis.
